# *Streptococcus suis* Research: Progress and Challenges

**DOI:** 10.3390/pathogens9090707

**Published:** 2020-08-27

**Authors:** Mariela Segura

**Affiliations:** Research Group on Infectious Diseases in Production Animals and Swine and Poultry Infectious Diseases Research Centre, Faculty of Veterinary Medicine, University of Montreal, St-Hyacinthe, QC J2S 2M2, Canada; mariela.segura@umontreal.ca

**Keywords:** *Streptococcus suis*, swine, zoonosis, epidemiology, genomics, diagnosis, antimicrobials, vaccines, public health

## Abstract

*Streptococcus suis* is considered among the top bacterial pathogens leading to important economic losses to the swine industry, with the incidence of disease increasing as the prophylactic use of antimicrobial is being vanished worldwide. *S. suis* is also a zoonotic agent afflicting people in close contact with infected pigs or pork meat. Besides, in some Asian countries, it is considered a major public health concern for the general population as well. Antimicrobial resistance is one of the most important global health challenges, and in the absence of preventive measures (such as effective vaccines), *S. suis* remains a risk for increased antimicrobial resistance and transmission of resistance genes to other bacteria beyond the host animal species. The studies in this Special Issue have evidenced the importance of swine population demographics and management on disease control, progress in molecular tools to better understand the epidemiology of *S. suis* infections in swine and humans, and the mechanisms involved in different aspects of the immuno-pathogenesis of the disease. The importance of reducing the prophylactic use of antimicrobials in livestock productions and the development of alternative control measures, including vaccination, are herein discussed.

Diseases caused by *Streptococcus suis* are a significant economic problem and an animal welfare concern in swine productions. In addition, this pathogen is a public health threat due to its high zoonotic potential [1]. In most Western countries, *S. suis* is considered an occupational disease affecting people working in the swine or pork industries. However, in Southeast Asian countries, the general population is at risk mainly due to close contact with animals or consumption of raw pork products [2,3,4]. Currently, 29 serotypes of *S. suis* are recognized based on antigenically different structures of the capsular polysaccharide (CPS) surrounding the bacterium [5]. Of these, serotype 2 is the most prevalent in *S. suis* swine and human infections, although human cases have been reported caused by serotypes 4, 5, 9, 14, 16, 21, 24, and 31 [4,6,7,8]. To better understand the dynamics influencing clinical disease in pigs and zoonotic infections, the studies published on this research topic (14 research articles, one review, and one conference report) have strengthened our knowledge on swine population demographics and the effect on disease, the epidemiology of *S. suis* infections in swine and humans, the mechanisms by which *S. suis* can invade and disseminate in the blood, and different aspects of host–pathogen interactions. Control strategies, and importantly, antimicrobial resistance and the role of *S. suis* in the spreading of antimicrobial resistance genes are discussed in the context of One Health.

*S. suis* is a disease related to swine production systems, and usually occurs in piglets up to 10 weeks of age. Indeed, *S. suis* is an early colonizer (animals are colonized during or soon after birth) and becomes part of the normal microflora, complicating eradication strategies [9]. The bacterium commonly resides in the upper respiratory tract of pigs and is genetically highly diverse. However, not all serotypes or all strains within a serotype have the potential to cause disease. Depending on partially uncovered conditions, such as environmental factors [1], pigs harboring *S. suis* may remain healthy carriers or develop clinical infection. Horizontal transmission of *S. suis* primarily occurs through the oro-nasal route, and aerosol transmission has also been proposed [10,11]. The introduction of carrier animals (harboring potentially pathogenic strains) into the herd has been suggested as one of the factors influencing outbreaks of disease, together with farm management practices in the nursery. Giang et al. [12] applied mathematical models for understanding the complex *S. suis* disease dynamics and to simulate disease control strategies that swine producers may employ to reduce *S. suis* spread in the nursery. They developed a stochastic mathematical model incorporating sub-clinically infected (“carriers”) pigs to capture the effects of changes in host recruitment rate on disease incidence. Results showed that monthly introduction of pigs into the nursery (instead of weekly introduction) reduces cumulative cases of *S. suis* by up to 59%, while increasing disease-removal rates alone averts up to 64% of cases. Findings suggest that modifications to host recruitment rates could be leveraged as a tool for *S. suis* disease control; however, further understanding disease transmission mechanisms can be critically important for implementing and optimizing disease control strategies [12].

As mentioned above, *S. suis* mainly affects post-weaned piglets and adult animals are generally considered resistant to the disease [1]. Nevertheless, Hennig-Pauka et al. [13] reported sudden death of fattening pigs kept under extensive husbandry conditions. The animals died suddenly of septic shock and showed disseminated intravascular coagulopathy. Isolated *S. suis* from dead pigs belonged to serotype 2 and was classified as sequence type (ST) 28. In this swine herd, pigs were kept under extensive husbandry conditions, ensuring a high animal welfare level with a low pig density and the freedom for pigs to carry out species-specific behavior. These husbandry conditions are completely different from the intensive husbandry found on conventional farms. Authors suggested that both insufficient exposure and interaction of the immune system in susceptible pigs with the pathogenic strain before the time point of infection, or intrinsic bacterial factors, might explain the outbreak in this farm [13]. Other pre-conditions for disease outbreak, including co-infections, also existed in these extensive husbandry conditions. Finally, transporting the pigs to the fattening site was a significant stressor, which coincided with exposure to a high bacterial load in the new environment due to a lack of adequate cleaning and disinfection measures [13]. This study stresses the importance of understanding disease dynamics and of applying appropriate management strategies to prevent this type of outbreaks.

Of the 29 serotypes, most *S. suis* isolates recovered from diseased pigs belong to serotypes 1 through 9, 1/2 and 14, though the distribution may differ depending on geographical location [1,4]. Nevertheless, serotypes 2, 9, and 3 predominate in Europe and Asia [4,14]. In North America, multiple serotypes have been recorded in diseased pigs, such as serotypes 2, 3, 1/2, 8, 4, and 7. To gain a better understanding of the complex epidemiology in this part of the globe, Denich et al. [15] performed a case-control study in Canada and observed that serotypes 9, 2, or 1/2 were the most commonly found in systemic sites of clinical cases. Detection of serotype 9 in upper respiratory sites was positively associated with their detection in systemic sites of clinical cases. However, no association between serotypes recovered from upper respiratory sites of cases and controls was detected. Un-typable isolates were also detected in high frequency. This study confirms that a variety of serotypes can be found in commercial swine productions in North America [15].

Information on the epidemiology of the infection in swine in South America is limited. Considering that Brazil is a major pork producer and exporter, Matajira et al. [16] characterized Brazilian *S. suis* strains isolated over a 15-year period from pigs with clinical signs of encephalitis, septicemia, arthritis, or pneumonia. Serotype prevalence revealed a predominance of serotype 2 (or 1/2), followed by 3, 7, 1 (or 14), 6, 8, 18, 28, and 27. The isolation of some uncommon serotypes might be explained by the inclusion of strains from the lungs of animals showing signs of pneumonia, where *S. suis* is considered an opportunistic pathogen rather that a primary cause of disease. The study also revealed that, independently of the isolation period, *S. suis* strains already presented high resistance to three and up to eight antimicrobial classes. The authors suggested that *S. suis* epidemiological surveillance must be reinforced in a large country such as Brazil, since multidrug resistance strains were identified at high rates. The human healthcare system must also be the subject of constant monitoring to evaluate whether the pathogen becomes a zoonotic risk in the region, where, until now, there are no cases registered of human *S. suis* infection [16].

Antimicrobial resistance is indeed one of the major global health challenges of this century, and *S. suis* is suspected to be a reservoir of antimicrobial resistance genes [17]. Libante et al. [18] screened the genomes of 214 strains of 27 serotypes for antimicrobial resistance genes and chromosomal Mobile Genetic Elements (MGEs), in particular Integrative Conjugative Elements (ICEs) and Integrative Mobilizable Elements (IMEs). This large-scale genome analysis revealed the high diversity of putative MGEs transferring by conjugation (ICEs and IMEs) in *S. suis*. In addition to the five families of ICEs already reported in *S. suis* (Tn5252, Tn1549, TnGBS2, Tn916, and vanG), ICEs of the ICESt3 and TnGBS1 families were also detected in a few strains. These families are frequent in other streptococci but were not reported in *S. suis* until this study. Almost 400 antimicrobial resistance genes were detected in the 214 genomes analyzed. More surprisingly, more than half of the detected antimicrobial resistance genes were carried by putative IMEs. These results suggest that besides ICEs, IMEs likely play a major role in the dissemination of antimicrobial resistance genes in *S. suis*. The work of Libante et al. [18] highlights the importance of reducing the prophylactic use of antimicrobials in livestock productions and the development of alternative control measures. In this regard, Wongsawan et al. [19] explored the bactericidal effect of clove oil against *S. suis* in vitro. It was found that eugenol is the major active ingredient of clove oil and a promising alternative product for control of infectious diseases caused by *S. suis*. However, the effectiveness of such a product in vivo remains to be evaluated.

Besides *S. suis’* impact on the swine industry, the number of reported human cases, especially in Southeast Asian countries, has considerably increased in the past years [4,6,20]. In this geographical area, poor quality of food safety controls for raw pork products at slaughterhouses and wet markets has been suggested as a source of infection in humans [21,22]. Kerdsin et al. [23] studied the prevalence and diversity of *S. suis* carriage in slaughterhouse pigs in Phayao province, Thailand, where a human outbreak occurred in 2007. *S. suis* carriage rate was high among slaughterhouse pigs and the prevalence rates of serotypes 2 and 14 (the major serotypes associated to human infections) were 6.7% and 2.6%, respectively. A large majority of these isolates revealed sequence types and pulso-types identical to human isolates in Thailand. The authors suggested that occupational exposure to pigs or the consumption of raw pork products are a risk factor. Food safety, hygiene, and health education should be encouraged to reduce the threat to humans [23].

As previously mentioned, *S. suis* colonizes the upper respiratory tract of pigs; therefore, respiratory epithelial cells represent a first barrier against *S. suis* infections. Nevertheless, the mechanisms used by *S. suis* strains to cross this mucosal line of defense are poorly known. Suilysin (SLY) is the key toxin of *S. suis* and it is a member of the family of cholesterol-dependent pore-forming toxins. SLY has been suggested to play a role in these early infection events [24,25,26]. Vötsch et al. [27] investigated the susceptibility of different respiratory epithelial cells to SLY, including immortalized cell lines (HEp-2 and NPTr cells), which are frequently used in in vitro studies addressing *S. suis* virulence mechanisms, as well as primary porcine respiratory cells. It was observed that the human epithelial cell line HEp-2 is the most susceptible, whereas primary porcine epithelial cells were hardly affected by the toxin. The results indicated that the amount of membrane-bound SLY, the cholesterol content of the cells, as well as their resealing capacity, all affect the susceptibility of the different cells to SLY effects. The authors suggested that the choice of cells for in vitro studies on biological functions of virulence-associated factors, such as SLY, should be performed with models which more closely represent the in vivo situation than permanent cell lines. The study of Vötsch et al. [27] also emphasized the importance of choosing the proper host species, as previous studies showed that SLY is a critical virulence factor in the mouse model, but not in pigs [28,29].

Several clinical manifestations caused by *S. suis* infection have been reported and linked to bacterial dissemination in blood, such as arthritis, endocarditis, and meningitis. Septicemia with sudden death (pigs) [1] or streptococcal toxic shock-like syndrome (humans) is a striking feature of this infection [30]. To survive and even proliferate in host blood, *S. suis* must mainly overcome the innate immunity and nutritional limitations in this host compartment [24]. Ma et al. [31] found that *S. suis* serotype 2 virulent strains with higher proliferative ability in swine serum than low-virulent strains, possess a >20 kb endoSS-related insertion region. Further analyses identified a complete N-glycans’ degradation system encoded within this insertion region. These findings provided evidence that the endoSS-related N-glycans’ degradation system enables *S. suis* serotype 2 to adapt to serum-specific availability of carbon sources, and it might be required for optimal colonization and full virulence during systemic infection. More studies will be required to confirm the presence of this system in other virulent *S. suis* strains and/or serotypes.

*S. suis*-associated pathologies are mainly the consequence of an exacerbated inflammatory response involving several cellular and molecular mediators, including pro-inflammatory cytokines [24]. Several bacterial components have been associated to either the down-modulation or the increased activation of different inflammatory pathways [24,32,33]. For example, high levels of SLY production were demonstrated to play a key role in *S. suis*-induced interleukin (IL)-1β production [34]. SLY is also involved in several other aspects of the bacterial–host interactions during *S. suis* dissemination in the host [26,35,36,37]. Nevertheless, not all *S. suis* virulent strains possess SLY [38]. In their study, Lavagna et al. [39] uncovered molecular mechanisms underlying the pathogenesis of the disease caused by SLY-negative strains. They showed that SLY-negative *S. suis* induces elevated levels of IL-1β in systemic organs, with dendritic cells contributing to this production. This production required recognition of bacterial lipoproteins by the Toll-like receptor 2 (TLR2)/Myeloid differentiation primary response 88 (MyD88) pathway. Nevertheless, the higher internalization rate of a SLY-negative strain resulted in intracellularly located DNA being recognized by the AIM2 (absent in melanoma 2) inflammasome, which promoted IL-1β production. In vivo experimental studies suggested that IL-1 might play a role in host survival during *S. suis* systemic infection, via modulation of the inflammation required to control bacterial burden [39].

In spite of numerous in vitro studies with mouse-, human- or swine-origin cells or in vivo studies using mouse models, in vivo data on the relationship between induced cytokines and bacteremia in pigs are missing. Hohnstein et al. [40] showed an increase in IL-6 and IL-10, but not in tumor necrosis factor (TNF)-α, IL-17A, or interferon (IFN)-γ levels, after experimental intravenous infection of piglets with *S. suis* serotype 2, which correlated with pronounced bacteremia during the first 24 h post-infection. Using an in vitro porcine whole-blood assay, it was shown that the *S. suis* serotype 2 strain and its non-encapsulated mutant induce similar levels of pro-inflammatory (i.e., IL-6) and anti-inflammatory (i.e., IL-10) cytokines in whole-blood. In addition, an early TNF-α induction was detected, as previously reported [41]. In contrast to the wild-type strain, the non-encapsulated mutant was rapidly eliminated in the whole-blood culture. However, when using peripheral blood mononuclear cells (PBMCs) and controlled bacteria:cell ratios, it could be observed that the CPS impairs cytokine production, as previously reported with other cell types [32]. Besides *S. suis* serotype 2, the study also reported similar levels and patterns of cytokine production by PBMCs stimulated with *S. suis* serotypes 7 and 9. Finally, the study of Hohnstein et al. [40] suggested that monocytes are the main producers of TNF-α in response to encapsulated *S. suis*; however, TNF-α does not contribute to bacterial killing in blood.

The two large-scale human outbreaks of streptococcal toxic shock-like syndrome in China suggest that the pathogenicity of *S. suis* has been changing in recent years. These outbreaks were caused by a serotype 2 and ST7 hyper-virulent clone, endemic to China only [42,43]. Genetic analysis revealed the presence of a unique chromosomal 89 kb pathogenicity island (later renamed SsPI-1) in Chinese epidemic *S. suis* strains [44,45]. Zhao et al. [46] assessed the impact of SsPI-1 deletion on the virulence of *S. suis*. Swine experimental results showed that elimination of SsPI-1 significantly impairs the pathogenicity of *S. suis*, suggesting that SsPI-1 plays an important role in bacterial virulence and disease-induced pathology. In addition, deletion of SsPI-1 reduced serum levels of TNF-α, IL-6, IL-8, and IL-1β in infected pigs, signifying either a direct role of SsPI-1 in the induction of pro-inflammatory cytokine responses within the host or an indirect effect because of reduced bacteremia. The authors concluded that SsPI-1 is a critical contributor to the evolution of virulence in epidemic *S. suis* [46]. Recently, Wang et al. [47] reported 38 sporadic ST7 *S. suis* strains, which mostly caused sepsis, from patients in the Guangxi Zhuang Autonomous Region between 2007 and 2018. Interestingly, among these sporadic ST7 strains, serotype 14 was the most frequent, followed by serotype 2. The analysis of the phylogenetic structure of the ST7 population, including epidemic and sporadic ST7 strains, revealed high diversity. Compared to the genome of the epidemic strain, the major differences in the genome of sporadic ST7 strains were the absence of the 89 kb SsPI-1 (specific to the epidemic strain) and the insertion of MGEs or ICE elements, suggesting a role in horizontal transfer of antibiotic resistance genes [18]. The emergence of these ST7 strains, and importantly within serotype 14 (reported by Wang et al. for the first time [47]), highlights the need to have a better understanding of the evolution of zoonotic ST7 strains and their ability to cause life-threatening infections in humans.

Global restrictions in prophylactic use of antimicrobials in livestock productions place vaccination as the most promising way to prevent *S. suis* infections. Nevertheless, no efficacious commercial vaccine is available in spite of numerous research studies on the subject [48]. Only autogenous vaccines are used in the field, with conflicting results [49]. In this regard, this Special Issue also included an update on policies and recommendations for the manufacture, control, and use of inactivated autogenous vaccines, to advance this important field of veterinary medicine (see Reference [50]).

Improving our knowledge of the adaptive immune response against this pathogen and potential target antigens will help advance vaccine development or improvement. Due to the presence of the thick CPS, *S. suis* is highly resistance to clearance by phagocytic cells unless there is a presence of opsonizing antibodies which facilitate bacterial elimination. Goyette-Desjardins et al. [51] characterized the protective activity of monoclonal antibodies (mAbs) directed against *S. suis* serotype 2 CPS. All mAbs targeted the sialylated chain of the CPS, but showed different cross-reactivity against *S. suis* serotypes 1, 1/2, and 14, whose CPS structures are similar to that of serotype 2 [52,53,54]. Only the immunoglobulin (Ig)G1 mAb was shown to be serotype 2-specific. Using an opsonophagocytosis assay, different IgM mAbs were opsonizing towards the *S. suis* serotypes to which they cross-react, while the IgG1 failed to induce bacterial elimination. However, in a model of mouse passive immunization, not all IgM mAbs were protective against a lethal challenge, indicating that both the Ig isotype and the target epitope influence the biological activity of the antibody. This study helps with defining the protective epitopes of *S. suis* serotype 2 CPS and provides fundamental knowledge to further advance the development of optimized vaccines against *S. suis*.

## Concluding Remarks

*S. suis* strain diversity is one of the major challenges that hampers progress towards the development of proper control strategies and epidemiological surveillance. The identification of molecular tools to differentiate potentially disease-associated from non-disease associated strains is of utmost importance, yet a goal that might be effortful to achieve. Hatrongjit et al. [55] reviewed recent advances in whole-genome analysis and other molecular approaches that can be used for surveillance, outbreak tracking, preventative health management, effective treatment and control, as well as vaccine development. In light of the zoonotic potential of *S. suis* and its re-emergence in the era of antimicrobial restriction, more research is urgently needed to fight this infection.
